# A Short Proof of Bose–Einstein Condensation in the Gross–Pitaevskii Regime and Beyond

**DOI:** 10.1007/s00023-024-01465-8

**Published:** 2024-07-01

**Authors:** Christian Brennecke, Morris Brooks, Cristina Caraci, Jakob Oldenburg

**Affiliations:** 1 Institute for Applied Mathematics, University of Bonn, Endenicher Allee 60, 53115 Bonn, Germany; 2 Institute for Mathematics, University of Zurich, Winterthurerstrasse 190, 8057 Zurich, Switzerland

## Abstract

We consider dilute Bose gases on the three-dimensional unit torus that interact through a pair potential with scattering length of order $N^{\kappa -1}$, for some $\kappa >0$. For the range $\kappa \in [0, \frac{1}{43})$, Adhikari et al. (Ann Henri Poincaré 22:1163–1233, 2021) proves complete BEC of low energy states into the zero momentum mode based on a unitary renormalization through operator exponentials that are quartic in creation and annihilation operators. In this paper, we give a new and self-contained proof of BEC of the ground state for $\kappa \in [0, \frac{1}{20})$ by combining some of the key ideas of Adhikari et al. (Ann Henri Poincaré 22:1163–1233, 2021) with the novel diagonalization approach introduced recently in Brooks (Diagonalizing Bose Gases in the Gross–Pitaevskii Regime and Beyond, arXiv:2310.11347), which is based on the Schur complement formula. In particular, our proof avoids the use of operator exponentials and is significantly simpler than Adhikari et al. (Ann Henri Poincaré 22:1163–1233, 2021).

## Introduction and Main Result

We consider *N* interacting bosons in $\Lambda := \mathbb {T}^3 = \mathbb {R}^3/ \mathbb {Z}^3$ with Hamiltonian

1 $$\begin{aligned} H_N = \sum _{i=1}^N -\Delta _{x_i} + \sum _{1\le i<j\le N} N^{2-2\kappa } V (N^{1-\kappa }(x_i -x_j)), \end{aligned}$$

acting in $L^2_s (\Lambda ^N)$, the Hilbert space consisting of functions in $L^2 (\Lambda ^N)$ that are invariant with respect to permutations of the *N* particles. We assume the interaction potential $V \in L^{1}(\mathbb {R}^3)$ to have compact support, to be radial and to be pointwise non-negative.

Note that analyzing $H_N$ is equivalent to analyzing the Hamiltonian of *N* bosons interacting through the unscaled potential *V* in $\mathbb {R}^3/ L\mathbb {Z}^3$ for $L=N^{1-\kappa }$. In this sense, we consider regimes of strongly diluted systems of bosons with number of particles density $N^{3\kappa -2} \ll 1$ (as long as $\kappa < \frac{2}{3}$). The case $\kappa =0$ corresponds to the Gross–Pitaevskii (GP) regime and the case $\kappa =\frac{2}{3}$ corresponds to the usual thermodynamic limit (with number of particles density equal to one).

In this paper, we are interested in understanding low energy properties of the Bose gas in regimes that interpolate between the GP and thermodynamic limits. Based on [20, 32], it is well-known that the ground state energy $E_N:= \inf \text {spec}(H_N)$ is equal to

$$\begin{aligned}E_N = 4\pi \mathfrak {a}N^{1+\kappa } +o(N^{1+\kappa }), \end{aligned}$$

where $\mathfrak {a}$ denotes the scattering length of the potential *V* and where $o(N^{1+\kappa })$ denotes an error of subleading order, that is, $\lim _{N\rightarrow \infty } o(N^{1+\kappa })/N^{1+\kappa }=0$. Recall that under our assumptions, the scattering length of *V* is characterized by

$$\begin{aligned}\begin{aligned} 8\pi \mathfrak {a}&= \inf \bigg \{ \int _{\mathbb {R}^3}dx\,\Big ( 2|\nabla f(x)|^2+V(x)|f(x)|^2\Big ): \lim _{|x|\rightarrow \infty }f(x)=1 \bigg \}. \end{aligned}\end{aligned}$$

A question closely related to the computation of the ground state energy is whether the ground state exhibits Bose–Einstein condensation (BEC). If $\psi _N$ denotes the ground state vector, this means that the largest eigenvalue of the associated reduced one particle density matrix $\gamma _N^{(1)} = \text {tr}_{2,\dots ,N} |\psi _N\rangle \langle \psi _N|$ remains of size one in the limit $N\rightarrow \infty$:

$$\begin{aligned}\liminf _{N\rightarrow \infty }\Vert \gamma _N^{(1)}\Vert _{\text {op}}>0.\end{aligned}$$

Proving BEC in the thermodynamic limit is a difficult open problem in mathematical physics. For strongly diluted systems, on the other hand, there has recently been great progress in proving that low energy states exhibit BEC. The first proof of BEC has been obtained in [28] in the GP regime,^1^ implying that for $\varphi _0:=1_{|\Lambda }\in L^2(\Lambda )$, one has that

2 $$\begin{aligned} \lim _{N\rightarrow \infty } \langle \varphi _0,\gamma _N^{(1)} \varphi _0\rangle =1. \end{aligned}$$

This result has later been extended to approximate ground states in [29, 35] and the works [5, 8] have proved (2) with the optimal rate of convergence. Since then, several generalizations and simplified proofs have been obtained in [1, 9, 13, 17, 21, 26, 33, 34]. Notice that such results can be used to derive the low energy excitation spectrum of $H_N$ in accordance with Bogoliubov theory [10], see e.g. [2, 7, 8, 11, 14, 16, 18, 19, 27, 36].

In recent years, progress has also been made in regimes that interpolate between the GP and thermodynamic limits. Based on unitary renormalizations developed first in the dynamical context [4, 12] and in the context of the derivation of the excitation spectrum in the GP regime [6, 7], the work [1] proves BEC for approximate ground states in regimes $\kappa \in [0,\frac{1}{43})$. A different method that is based on box localization arguments has been introduced in [21] which proves BEC in the larger parameter range $\kappa \in [0, \frac{2}{5}+\epsilon )$, for some sufficiently small $\epsilon >0$. This result represents currently the best available parameter range and it is closely tied to the computation of the second-order correction to the ground state energy, which turns out to be of order $N^{ 5\kappa /2}$ [3, 23–25, 37].

The methods introduced in [1, 21] have both certain advantages. While [21] obtains the currently best parameter range and applies to a large class of potentials including hard-core interactions, it is based on box localization arguments and therefore involves the change of boundary conditions.^2^ This makes the derivation of suitable lower bounds more complicated, compared to the translation invariant setting, and essentially restricts the method to obtaining lower bounds while upper bounds require separate tools. The method of [1], on the other hand, does not require localization and enables both upper and lower bounds at the same time. However, it only applies to soft potentials satisfying some mild integrability assumption. Moreover, controlling the error terms in the operator expansions quickly becomes rather challenging and this is among the main reasons why the method only works in a much more restricted parameter range.

In this paper, our goal is to revisit the strategy of [1]. However, instead of renormalizing the system through unitary conjugations by quartic operator exponentials, we proceed as in [16] whose renormalization is based on the Schur complement formula applied to the two body problem and on lifting it in a suitable sense to the *N* body setting. As a consequence, our proof becomes significantly simpler and shorter compared to the one in [1]. Although our results are still only valid in a small parameter range compared to [21], our arguments are elementary, self-contained and do neither require box localization methods nor operator exponential expansions.

### Theorem 1

Let $H_N$ be defined as in (1) for $\kappa \in [0, \frac{1}{20})$ and denote by $\gamma _N^{(1)}$ the one particle reduced density associated with its normalized ground state vector $\psi _N$. Then,

$$\begin{aligned}\lim _{N\rightarrow \infty } \langle \varphi _0,\gamma _N^{(1)} \varphi _0\rangle =1. \end{aligned}$$

### Remark

1. Theorem 1 applies to the ground state vector $\psi _N$ of $H_N$. With some additional effort that involves the use of number of particles localization arguments, we expect that our results could also be proved for approximate ground states $\phi _N$ that satisfy $\langle \phi _N, H_N\phi _N\rangle \le 4\pi \mathfrak {a}N^{1+\kappa }+ o(N)$. To keep our arguments as short and simple as possible, we omit the details and focus on the ground state vector $\psi _N$.
2. In our proof of Theorem 1, we assume the relatively mild a priori information that the ground state energy $$ E_N $$ is bounded from above by $$ E_N \le 4\pi \mathfrak {a}N^{1+\kappa }+ o(N)$$, if $$\kappa <\frac{1}{20}$$. Based on ideas similar to those presented below, this could be proved with little additional effort in a self-contained way. Since this has already been explained in [16] (which obtains a more precise upper bound on $$ E_N$$ for all $$\kappa <\frac{2}{13}$$ based on the evaluation of the energy of suitable trial states, see [16, Theorem 3]), however, we refer the interested reader to [16] for the details.

## Proof of Theorem 1

In the following, let us denote by $$ a_k$$ and $$a^*_k$$ the annihilation and, respectively, creation operators associated with the plane waves $$ x\mapsto \varphi _k(x):=e^{ikx} \in L^2(\Lambda )$$ of momentum *k*, for $$k\in \Lambda ^*:=2\pi \mathbb {Z}^3$$. They satisfy the canonical commutation relations $$ [a_p, a_q^*] = \delta _{p,q}$$ and $$ [a_p,a_q]=[a_p^*, a_q^*]=0$$, and they can be used to express $$ H_N$$ as

$$\begin{aligned} H_N = \sum _{r\in \Lambda ^*}|r|^2 a^*_ra_r + \frac{N^{\kappa }}{2N}\sum _{p,q,r\in \Lambda ^*} \widehat{V}(r/N^{1-\kappa }) a^*_{p+r}a^*_{q-r}a_pa_q, \end{aligned}$$

where $\widehat{V}(r) = \int _{\mathbb {R}^3}dx\, e^{-irx}V(x)$ denotes the standard Fourier transform of *V*.

Now, denote by $$ V_N$$ the two body operator that multiplies by $$N^{2-2\kappa }V(N^{1-\kappa }(x_1-x_2)) $$ in $$ L^2(\Lambda ^2)$$ and define for $$\alpha \in [0, 1-\kappa ]$$ the low-momentum set

3 $$\begin{aligned} \text {P}_{\text {L}} := \big \{p\in \Lambda ^*: |p|\le N^{\alpha } \big \}. \end{aligned}$$

Denote, moreover, by $\Pi _{\text {L}} :L^2(\Lambda ^2)\rightarrow L^2(\Lambda ^2)$ the orthogonal projection onto

$$\begin{aligned} \overline{ \text {span}(\varphi _k\otimes \varphi _l:k,l\in \text {P}_{\text {L}} )} \end{aligned}$$

and set $\Pi _{\text {H}} :=1-\Pi _{\text {L}}$. Then, as explained in detail in [16], a straightforward application of the Schur complement formula implies the many body lower bound

4 $$\begin{aligned} H_N \ge \sum _{r\in \Lambda _+^*}|r|^2 c^*_r c_r + \frac{N^{\kappa }}{2N} \sum _{\begin{array}{c} p,q,r\in \Lambda ^*: \\ p,q, p+r, q-r \in \text {P}_{\text {L}} \end{array}} \langle \varphi _{p+r}\otimes \varphi _{q-r}, V_{\text {ren}}\varphi _p\otimes \varphi _q \rangle a^*_{p+r}a^*_{q-r}a_pa_q - R_N, \nonumber \\ \end{aligned}$$

where we set $\Lambda _+^*:= \Lambda ^*{\setminus }\{0\}$ as well as

5 $$\begin{aligned} \begin{aligned}&c_r := a_r + \frac{N^{\kappa }}{N}\sum _{\begin{array}{c} (p,q)\in \text {P}_{\text {L}} ^2 \end{array}} \langle \varphi _{p+q-r}\otimes \varphi _{r}, \eta \, \varphi _p\otimes \varphi _q \rangle a^*_{p+q-r}a_pa_q,\\&\eta := N^{1-\kappa }\,\Pi _{\text {H}} \big [ \Pi _{\text {H}} (-\Delta _{x_1}-\Delta _{x_2} + V_N )\Pi _{\text {H}} \big ]^{-1}\Pi _{\text {H}} V_N\Pi _{\text {L}} ,\\&V_{\text {ren}}:= N^{1-\kappa }\big ( V_N - V_N \Pi _{\text {H}} \big [ \Pi _{\text {H}} (-\Delta _{x_1}-\Delta _{x_2} + V_N )\Pi _{\text {H}} \big ]^{-1}\Pi _{\text {H}} V_N \big ), \end{aligned}\end{aligned}$$

and where the three body error term $R_N$ is given by

6 $$\begin{aligned} R_N&:= \frac{N^{2\kappa }}{N^2} \sum \limits _{\begin{array}{c} r, p,q, s, t\in \Lambda ^* \end{array}} |r|^2 \langle \eta \,\varphi _p\otimes \varphi _q, \varphi _{p+q-r}\otimes \varphi _{r} \rangle \langle \varphi _{s+t-r}\otimes \varphi _{r}, \eta \, \varphi _{s}\otimes \varphi _{t} \rangle \nonumber \\&\quad \times a_p^*a^*_q a^*_{s+t-r}a_{p+q-r}a_{s}a_{t}. \end{aligned}$$

Notice that we used that both $\eta$ and $V_{\text {ren}}$ preserve the total momentum in $L^2(\Lambda ^2)$.

Let us briefly comment on the main ideas leading to (4). Viewing $V_N = \Pi _{\text {L}} V_N \Pi _{\text {L}} + (\Pi _{\text {H}} V_N \Pi _{\text {L}} +\text {h.c.}) + \Pi _{\text {H}} V_N\Pi _{\text {H}}$ and hence the Hamiltonian $\mathcal {H}_2:=-\Delta _{x_1}-\Delta _{x_2}+V_N$ of the two body problem as a block matrix, one can block-diagonalize the latter using the Schur complement formula. This renormalizes the low-momentum interaction to $N^{\kappa -1} \Pi _{\text {L}} V_{\text {ren}}\Pi _{\text {L}}$, while the large momentum interaction $\Pi _{\text {H}} V_N\Pi _{\text {H}}$ is left untouched. The (non-symmetric) map that block-diagonalizes $\mathcal {H}_2$ is of the form $\mathcal {S}_\eta = 1+N^{\kappa -1}\eta$ and, in order to obtain an analogous renormalization of the many body interaction, it seems natural to lift $\mathcal {S}_\eta$ to the unitary generalized Bogoliubov transformation

$$\begin{aligned}\begin{aligned}&\mathcal {U}_\eta := \exp (\mathcal {D}_\eta -\mathcal {D}_\eta ^*)\, \big (\approx 1+ \mathcal {D}_\eta -\mathcal {D}_\eta ^*\big ), \text { where }\\&\mathcal {D}_\eta := \frac{N^{\kappa }}{2N}\sum _{\begin{array}{c} p,q,r\in \Lambda ^*: (p,q)\in \text {P}_{\text {L}} ^2,\\ (p-r,q+r)\in ( \text {P}_{\text {L}} ^2)^c \end{array}}\langle \varphi _{p-r}\otimes \varphi _{q+r}, \eta \, \varphi _p\otimes \varphi _q \rangle a^*_{p-r}a^*_{q+r} a_pa_q. \end{aligned} \end{aligned}$$

On a conceptual level, this approach corresponds to the one pursued in [1] (in particular, the role of $\eta$ defined in (5) is similar to that of $\eta _{\text{ H }}$ defined in [1] through the zero energy scattering equation). Compared to that a key idea of [16] is to expand $H_N$ directly around powers of suitably modified creation and annihilation operators, including e.g. $c_r = a_r + [a_r, \mathcal {D}_\eta ] \,(\approx \mathcal {U}_\eta ^* a_r\,\mathcal {U}_\eta$). This leads to the low-momentum renormalization of the many body interaction in a simple way and avoids the use of operator exponential expansions. Notice that this approach is reminiscent of previously introduced ideas in [15, 23]. Finally, let us stress that, although the bound (4) is all we need in view of Theorem 1, Brooks [16] derives in fact exact algebraic identities. Similarly as in [1], what is dropped in (4) is the non-renormalized high momentum part of the potential energy.

Proceeding as in [16, Lemma 1], let us record the useful upper bounds

7 $$\begin{aligned} \begin{aligned}&\big |\langle \varphi _{k_1}\otimes \varphi _{k_2}, V_{\text {ren}}\varphi _{k_3}\otimes \varphi _{k_4} \rangle \big |\le C, \\&\big |\langle \varphi _{k_1}\otimes \varphi _{k_2}, V_{\text {ren}}\varphi _{k_3}\otimes \varphi _{k_4} \rangle - 8\pi \mathfrak {a} \big | \! \le C N^{\kappa -1 } \left( N^{\alpha } + \sum _{i=1}^4 N^{-\alpha } |k_i|^2\right) , \end{aligned}\end{aligned}$$

for all $k_1,k_2,k_3,k_4\in \Lambda ^*$ satisfying $k_1+k_2=k_3+k_4$ and $\langle \varphi _{k_1}\otimes \varphi _{k_2}, V_{\text {ren}}\varphi _{k_3}\otimes \varphi _{k_4} \rangle =0$ in case $k_1+k_2\ne k_3+k_4$. The bounds (7) imply in particular that

8 $$\begin{aligned}{} & {} \big |\langle \varphi _{k_1}\otimes \varphi _{k_2}, \eta \, \varphi _{k_3}\otimes \varphi _{k_4} \rangle \big |\le \frac{C\,\delta _{k_1+k_2,k_3+k_4}}{|k_1|^2+ |k_2|^2} {\textbf {1}}_{( \text {P}_{\text {L}} ^2)^c}((k_1,k_2)){\textbf {1}}_{ \text {P}_{\text {L}} ^2}((k_3,k_4)). \end{aligned}$$

For completeness, we prove (7) and (8) in Appendix A, following [16, Appendix A].

Based on (4), (7) and (8), the proof of Theorem 1 follows by carefully estimating the three terms on the r.h.s. in (4) and by combining these estimates with some mild a priori information on the ground state energy. Before summarizing the key steps, let us introduce the following additional notation: for every $\zeta \ge 0$, we set

$$\begin{aligned} \mathcal {N}_{>\zeta }:= \sum _{r\in \Lambda ^*: |r|>\zeta } a^*_ra_r \end{aligned}$$

and similarly, we define $\mathcal {N}_{\ge \zeta }, \mathcal {N}_{<\zeta }$ and $\mathcal {N}_{\le \zeta }$. Moreover, we set $\mathcal {N}:=\mathcal {N}_{\ge 0}\ (\equiv N)$, $\mathcal {N}_+:=\mathcal {N}_{>0}$ and $\mathcal {K}:= \sum _{r\in \Lambda ^*_+}|r|^2a^*_ra_r$. It is an elementary observation that

$$\begin{aligned}1-\langle \varphi _0, \gamma _N^{(1)}\varphi _0\rangle = N^{-1} \langle \psi _N,\mathcal {N}_+\psi _N\rangle .\end{aligned}$$

Equipped with the previous identity, the key of our proof is to derive a coercivity bound

$$\begin{aligned} H_N \ge 4\pi \mathfrak {a} N^{1+\kappa } + c\, \mathcal {N}_+ + \mathcal {E}\end{aligned}$$

for some constant $c>0$ and some error $\mathcal {E}$ which is of size *o*(*N*) in the ground state $\psi _N$. The number of excitations $\mathcal {N}_+$ is extracted from the modified kinetic energy operator in (4) (the first term on the r.h.s. in (4)) while the leading order energy $4\pi \mathfrak {a} N^{1+\kappa }$ is extracted from the renormalized potential energy (the second term on the r.h.s. in (4)). This is explained in Lemmas 2 and 3 which represent the key of the whole argument.

The error terms, on the other hand, turn all out to be related to the number of excitations with large momenta. Following [1], the key tool we use below to control such errors is a simple Markov bound combined with the trivial fact that $E_N\le C N^{1+\kappa }$:

9 $$\begin{aligned} \mathcal {N}_{ > N^{\beta }}\le N^{-2\beta }\mathcal {K}\le N^{-2\beta }H_N. \end{aligned}$$

In particular $\langle \psi _N, \mathcal {N}_{ > N^{\beta }}\psi _N \rangle \le C N^{1+\kappa -2\beta } = o(N)$ as soon as $2\beta >\kappa$, if $\psi _N$ denotes an approximate ground state vector. In Lemma 5, we slightly generalize the bound (9) to products of the kinetic energy with number of particles operators for large momenta.

### Lemma 2

Suppose $\delta \in (\frac{\kappa }{2},\alpha )$, then we have that

$$\begin{aligned}\sum _{r\in \Lambda _+^*}|r|^2 c^*_r c_r \ge 4\pi ^2 \big (\mathcal {N}_{< N^{ \delta }} -a^*_0a_0 \big ) + \mathcal {E}_\delta \end{aligned}$$

for a self-adjoint operator $\mathcal {E}_\delta$ which satisfies for some $C>0$ and *N* large enough that

$$\begin{aligned} \pm \mathcal {E}_{\delta } \le CN^{\kappa +\frac{\delta }{2}-\frac{3\alpha }{2}-1} (\mathcal {K}+ N )\mathcal {N}_{> N^\alpha /3}. \end{aligned}$$

### Proof

Recalling the definition of $c_r$ in (5) and setting

$$\begin{aligned}d_r = \frac{N^{\kappa }}{N}\sum _{\begin{array}{c} (p,q)\in \text {P}_{\text {L}} ^2 \end{array}} \langle \varphi _{p+q-r}\otimes \varphi _{r}, \eta \, \varphi _p\otimes \varphi _q \rangle a^*_{p+q-r}a_pa_q,\end{aligned}$$

so that $c_r = a_r + d_r$, we lower bound

$$\begin{aligned}\begin{aligned}&\sum _{r\in \Lambda _+^*}|r|^2 c^*_r c_r -4\pi ^2 \big (\mathcal {N}_{< N^{ \delta }} -a^*_0a_0 \big )\\&\quad \ge \sum _{r\in \Lambda _+^*:0< |r|< N^\delta }4\pi ^2 c^*_r c_r -\sum _{r\in \Lambda _+^*:0< |r|< N^\delta } 4\pi ^2a^*_r a_r\\&\quad = \sum _{r\in \Lambda _+^*:0< |r|< N^\delta } 4\pi ^2\big ( d^*_ra_r +a^*_r d_r + d^*_r d_r\big )\\&\quad \ge \frac{4\pi ^2N^{\kappa }}{N} \!\!\!\!\!\!\!\sum _{p,q, r\in \Lambda ^*: 0< |r| < N^\delta } \!\!\! \langle \varphi _{p+q-r}\otimes \varphi _{r}, \eta \, \varphi _p\otimes \varphi _q \rangle a^*_r a^*_{p+q-r}a_pa_q +\text {h.c.}, \end{aligned} \end{aligned}$$

where in the first and last steps, we used the positivity of $c^*_rc_r\ge 0$ and $d^*_rd_r\ge 0$, respectively. With the bound (8) and Cauchy–Schwarz, we then obtain for $\xi \in L^2_s(\Lambda ^N)$

$$\begin{aligned}&\bigg | N^{\kappa -1} \sum _{p,q, r\in \Lambda ^*: 0< |r|< N^\delta } \langle \varphi _{p+q-r}\otimes \varphi _{r}, \eta \, \varphi _p\otimes \varphi _q \rangle \langle \xi a^*_r a^*_{p+q-r}a_pa_q \xi \rangle \bigg | \\&\quad \le C N^{\kappa -2\alpha -1}\!\!\!\sum _{\begin{array}{c} (p,q,r)\in P_L^3: \\ 0< |r| < N^\delta ,\, |p|> N^\alpha /3,\, p+q-r\in \text {P}_{\text {L}} ^c \end{array}} \frac{|r|}{|q|+1}\Vert a_r a_{p+q-r}\xi \Vert \frac{|q|+1}{|r|}\Vert a_p a_q \xi \Vert \\&\quad \le CN^{\kappa +\frac{\delta }{2}-\frac{3\alpha }{2}-1} \langle \xi , (\mathcal {K}+ N )\mathcal {N}_{> N^\alpha /3}\xi \rangle . \end{aligned}$$

Notice that due to the constraint $p+q-r \in \text {P}_{\text {L}} ^c$ and the condition $|r| < N^\delta$ for $\delta < \alpha$, at least one of the momenta *p* and *q* has to be larger than $N^\alpha /3$ for large *N*. $\square$

### Lemma 3

There exists a constant $C>0$ such that

10 $$\begin{aligned}{} & {} \frac{N^{\kappa }}{2N}\!\!\!\sum _{\begin{array}{c} p,q,r\in \Lambda ^*: \\ p,q, p+r, q-r \in \text {P}_{\text {L}} \end{array}}\!\!\!\!\! \langle \varphi _{p+r}\otimes \varphi _{q-r}, V_{ren } \varphi _p\otimes \varphi _q \rangle a^*_{p+r}a^*_{q-r}a_pa_q \nonumber \\{} & {} \quad \ge 4\pi \mathfrak {a} N^{1+\kappa } - CN^\kappa \mathcal {N}_{>N^\alpha } -CN^{\kappa +3\alpha } - C N^{2\kappa +2\alpha -1} (\mathcal {K}+ N) \nonumber \\ \end{aligned}$$

### Proof

We use the bound (7) together with the fact that $|p|, |q|, |r|\le 2 N^\alpha$ if $p,q,p-r,q+r\in \text {P}_{\text {L}}$ to replace $V_{\text {ren}}$ as follows: For every $\xi \in L^2_s(\Lambda ^N)$, we have that

$$\begin{aligned}\begin{aligned}&\frac{N^{\kappa }}{2N}\!\sum _{\begin{array}{c} p,q,r \in \Lambda ^*:\\ p, q, p+r, q-r \in P_L \end{array}}\! \big | \langle \varphi _{p+r}\otimes \varphi _{q-r}, V_{\text {ren}}\varphi _p\otimes \varphi _q \rangle -8\pi \mathfrak {a}\big | |\langle \xi , a^*_{p+r}a^*_{q-r}a_pa_q\xi \rangle |\\&\quad \le C N^{2\kappa + \alpha - 2}\!\sum _{\begin{array}{c} p,q,r \in \Lambda ^*:\\ p, q, p+r, q-r \in P_L \end{array}}\! \frac{|p+r|+1}{|p|+1}\Vert a_{p+r}a_{q-r}\xi \Vert \frac{|p|+1}{|p+r|+1}\Vert a_pa_q\xi \Vert \\&\quad \le C N^{2\kappa +2\alpha -1} \langle \xi , (\mathcal {K}+ N) \xi \rangle . \end{aligned}\end{aligned}$$

As a consequence, we get the lower bound

$$\begin{aligned}\begin{aligned}&\frac{N^{\kappa }}{2N}\!\!\!\sum _{\begin{array}{c} p,q,r\in \Lambda ^*: \\ p,q, p+r, q-r \in \text {P}_{\text {L}} \end{array}}\!\!\!\!\! \langle \varphi _{p+r}\otimes \varphi _{q-r}, V_{ren } \varphi _p\otimes \varphi _q \rangle a^*_{p+r}a^*_{q-r}a_pa_q \\&\quad \ge \frac{4\pi \mathfrak {a} N^{\kappa }}{N}\!\!\!\sum _{\begin{array}{c} p,q,r\in \Lambda ^*: \\ p,q, p+r, q-r \in \text {P}_{\text {L}} \end{array}}\!\!\!\!\! a^*_{p+r}a^*_{q-r}a_pa_q - C N^{2\kappa +2\alpha -1} (\mathcal {K}+ N). \end{aligned}\end{aligned}$$

The lemma now follows by combining this estimate with the lower bound

$$\begin{aligned}\begin{aligned}&\frac{4\pi \mathfrak {a} N^{\kappa }}{N}\!\!\!\sum _{\begin{array}{c} p,q,r\in \Lambda ^*: \\ p,q, p+r, q-r \in \text {P}_{\text {L}} \end{array}}\!\!\!\!\! a^*_{p+r}a^*_{q-r}a_pa_q \\&\quad = \frac{4\pi \mathfrak {a} N^{\kappa }}{N} \sum _{r\in \Lambda ^*} \bigg (\sum _{\begin{array}{c} q\in \text {P}_{\text {L}} :\\ q+r\in \text {P}_{\text {L}} \end{array}} a^*_{q}a_{q+r}\bigg )^* \bigg (\sum _{\begin{array}{c} q\in \text {P}_{\text {L}} :\\ q+r\in \text {P}_{\text {L}} \end{array}} a^*_{q}a_{q+r}\bigg ) - \frac{4\pi \mathfrak {a} N^{\kappa }}{N}\!\!\!\sum _{\begin{array}{c} p,r\in \Lambda ^*: \\ p, p+r \in \text {P}_{\text {L}} \end{array}}\!\!\!\!\! a^*_{p+r} a_{p+r}\\&\quad \ge \frac{4\pi \mathfrak {a} N^{\kappa }}{N} \bigg (\sum _{\begin{array}{c} q\in \text {P}_{\text {L}} \end{array}} a^*_{q}a_{q}\bigg )^* \bigg (\sum _{\begin{array}{c} q\in \text {P}_{\text {L}} \end{array}} a^*_{q}a_{q}\bigg ) - \frac{4\pi \mathfrak {a} N^{\kappa }}{N}\!\!\!\sum _{\begin{array}{c} p,r\in \Lambda ^*: \\ p, p+r \in \text {P}_{\text {L}} \end{array}}\!\!\!\!\! a^*_{p+r} a_{p+r}\\&\quad = \frac{4\pi \mathfrak {a} N^{\kappa }}{N}\big (N- \mathcal {N}_{>N^{\alpha }}\big )^2- \frac{4\pi \mathfrak {a} N^{\kappa }}{N}\!\!\!\sum _{\begin{array}{c} p,r\in \Lambda ^*: \\ p, p+r \in \text {P}_{\text {L}} \end{array}}\!\!\!\!\! a^*_{p+r} a_{p+r}\\&\quad \ge 4\pi \mathfrak {a} N^{1+\kappa } - 8\pi \mathfrak {a} N^{\kappa }\mathcal {N}_{>N^{\alpha }} - C N^{\kappa +3\alpha }, \end{aligned}\end{aligned}$$

where in the last step we dropped the positive contribution proportional to $\mathcal {N}_{>N^{\alpha }}^2$ and where we used that $\mathcal {N}_{\le N^{\alpha }}\le N$ as well as $| \text {P}_{\text {L}} |\le CN^{3\alpha }$. $\square$

### Lemma 4

Let $R_N$ be as in (6) and let $0\le \beta < \alpha$. Then, there exists $C>0$ such that for *N* large enough, we have that

$$\begin{aligned}\begin{aligned} {\pm } R_N&\le CN^{2\kappa -2\alpha -2} \Big (N^{4\alpha } (\mathcal {N}_{>N^{\beta }}+N^{3\alpha }) + N^{\frac{5}{2}\alpha + \frac{3}{2}\beta +\frac{1}{2}} (\mathcal {N}_{>N^{\beta }}+N^{3\alpha })^{\frac{1}{2}} +N^{\frac{3}{2}\alpha +\frac{5}{2}\beta +1}\Big )\\&\quad \times \big (\mathcal {K}+ N + N^{5\beta }\big ) \big (\mathcal {N}_{>N^{\alpha }/3}+1\big ). \end{aligned}\end{aligned}$$

### Proof

Given $\xi \in L^2_s(\Lambda ^N)$, we apply the bound (8) to get

$$\begin{aligned}\begin{aligned} |\langle \xi , R_N\xi \rangle |&\le \frac{CN^{2\kappa }}{N^2}\!\!\!\sum _{\begin{array}{c} r\in \Lambda ^*_+, p,q, s, t\in \Lambda ^*:\\ (p+q-r,r),(s+t-r,r)\in ( \text {P}_{\text {L}} ^2)^c,\\ (p,q),(s,t)\in \text {P}_{\text {L}} ^2 \end{array}} \!\!\! \frac{|r|^2 | \langle \xi , a_p^* a_q^* a_{s+t-r}^* a_{p+q-r}a_{s}a_{t}\xi \rangle |}{(|p+q-r|^2+ |r|^2)(|s+t-r|^2+ |r|^2)}\\&\le CN^{2\kappa -2\alpha -2} \!\!\!\sum _{\begin{array}{c} r\in \Lambda ^*_+, p,q, s, t\in \Lambda ^*:\\ (p+q-r,r),(s+t-r,r)\in ( \text {P}_{\text {L}} ^2)^c,\\ (p,q),(s,t)\in \text {P}_{\text {L}} ^2 \end{array}} \!\!\! |\langle \xi , a_p^* a_q^* a_{s+t-r}^* a_{p+q-r}a_{s}a_{t}\xi \rangle |. \end{aligned}\end{aligned}$$

In order to control the sum on the right-hand side, we split it according to two types of restrictions: First, consider another scale $N^{\beta }$, for $\beta < \alpha$, and consider the cases in which the momenta $p,q,s,t\in \text {P}_{\text {L}} ^4$ are smaller or greater than $N^\beta$. We consider the cases

11 $$\begin{aligned} \begin{aligned}&(1)\;\;|p|, |q|, |s|, |t| \le N^\beta ,\\&(3)\;\;|p|, |q|> N^\beta \text { and } |s|, |t| \le N^\beta , \\&(5)\;\;|p|, |q|, |s|> N^\beta \text { and } |t| \le N^\beta , \end{aligned} \hspace{1cm} \begin{aligned}&(2)\;\;|p|> N^\beta \text { and } |q|, |s|, |t| \le N^\beta ,\\&(4)\;\;|p|, |s|> N^\beta \text { and } |q|, |t| \le N^\beta ,\\&(6)\;\; |p|, |q|, |s|, |t| > N^\beta . \end{aligned}\nonumber \\ \end{aligned}$$

Furthermore, the conditions $$(p+q-r,r), (s+t-r,r)\in ( \text {P}_{\text {L}} ^2)^c$$ imply that at least one of $$p, q, p+q-r$$ and one of $$ s,t, s+t-r$$ is greater than $$N^\alpha /3$$: we consider the cases

12 $$\begin{aligned} \begin{aligned}&(a)\;\;|p+q-r|, |s+t-r|> N^\alpha /3, \\&(c)\;\; |p+q-r|, |s|> N^\alpha /3, \end{aligned} \hspace{1cm} \begin{aligned}&(b)\;\;|p|, |s+t-r|> N^\alpha /3,\\&(d)\;\;|p|, |s|> N^\alpha /3. \end{aligned}\nonumber \\ \end{aligned}$$

Now, using symmetries among and within the pairs $(p,q)\in \text {P}_{\text {L}} ^2$ and $(s,t)\in \text {P}_{\text {L}} ^2$, one readily sees that for N large enough, such that $N^\beta < N^\alpha /3$, we have that

$$\begin{aligned}\begin{aligned}&\sum _{\begin{array}{c} r\in \Lambda ^*_+, p,q, s, t\in \Lambda ^*:\\ (p+q-r,r),(s+t-r,r)\in ( \text {P}_{\text {L}} ^2)^c,\\ (p,q),(s,t)\in \text {P}_{\text {L}} ^2 \end{array}} \!\!\! |\langle \xi , a_p^* a_q^* a_{s+t-r}^* a_{p+q-r}a_{s}a_{t}\xi \rangle |\\&\quad \le C \bigg ( \sum _{j=1}^6\Sigma _{ja}(\xi ) + \sum _{j=2}^6\Sigma _{jb}(\xi ) + \Sigma _{5c}(\xi ) +\sum _{j=4}^6\Sigma _{jd}(\xi )\bigg ), \end{aligned}\end{aligned}$$

where $\Sigma _{j\alpha }$, for $j\in \{1,\ldots , 6\}$ and $\alpha \in \{a,b,c,d\}$, refers to the contribution

$$\begin{aligned}\Sigma _{j\alpha }(\cdot ):=\sum _{\begin{array}{c} r\in \Lambda ^*_+, p,q, s, t\in \Lambda ^*: p,q,s,t,\\ p+q-r,s+t-r \text { satisfy } j) \text { and } \alpha ) \end{array}} \!\!\! |\langle \cdot , a_p^* a_q^* a_{s+t-r}^* a_{p+q-r}a_{s}a_{t}\cdot \rangle |\ge 0. \end{aligned}$$

Here, the restriction labels $j\in \{1,\ldots , 6\}$ and $\alpha \in \{a,b,c,d\}$ refer to (11) and (12), respectively. Applying basic Cauchy–Schwarz estimates as in Lemmas 2 and 3, we find

$$\begin{aligned}\begin{aligned}&\Sigma _{1a} \le C N^{4\beta +1} (\mathcal {K}+ N) (\mathcal {N}_{>N^{\alpha }/3}+1),\\&\Sigma _{2a},\Sigma _{3a}\le CN^{2\alpha + 2\beta +\frac{1}{2}} (\mathcal {K}+ N) (\mathcal {N}_{>N^{\alpha }/3}+1) (\mathcal {N}_{>N^{\beta }}+1)^{\frac{1}{2}},\\&\Sigma _{2b} \le CN^{\frac{3}{2}\alpha +\frac{5}{2}\beta +1} (\mathcal {K}+ N) (\mathcal {N}_{>N^{\alpha }/3}+1),\\&\Sigma _{3b} \le C\big ( N^{\frac{3}{2}\alpha + \frac{5}{2}\beta +\frac{1}{2}} (\mathcal {N}_{>N^{\beta }}+N^{3\alpha })^{\frac{1}{2}} + N^{3\alpha +\beta } (\mathcal {N}_{>N^{\beta }}+N^{3\alpha })\big )\\&\qquad \quad \times (\mathcal {K}+ N + N^{5\beta }) (\mathcal {N}_{>N^{\alpha }/3}+1),\\&\Sigma _{4a},\Sigma _{5a},\Sigma _{6a} \le C N^{4\alpha } (\mathcal {K}+ N) (\mathcal {N}_{>N^{\alpha }/3}+1) (\mathcal {N}_{>N^{\beta }}+1),\\&\Sigma _{4b},\Sigma _{5b},\Sigma _{6b} \le C\big ( N^{\frac{5}{2}\alpha + \frac{3}{2}\beta +\frac{1}{2}} (\mathcal {N}_{>N^{\beta }}+1)^{\frac{1}{2}} + N^{4\alpha } (\mathcal {N}_{>N^{\beta }}+1)\big ) (\mathcal {K}+ N) (\mathcal {N}_{>N^{\alpha }/3}+1), \\&\Sigma _{5c} \le C\big ( N^{2\alpha + 2\beta +\frac{1}{2}} (\mathcal {N}_{>N^{\beta }}+1)^{\frac{1}{2}} +N^{\frac{7}{2}\alpha +\frac{1}{2}\beta } (\mathcal {N}_{>N^{\beta }}+1)\big ) (\mathcal {K}+ N) (\mathcal {N}_{>N^{\alpha }/3}+1), \\&\Sigma _{4d}, \Sigma _{5d}, \Sigma _{6d} \le C\big ( N^{\frac{5}{2}\alpha + \frac{3}{2}\beta +\frac{1}{2}} (\mathcal {N}_{>N^{\beta }}+1)^{\frac{1}{2}} +N^{4\alpha } (\mathcal {N}_{>N^{\beta }}+1) +N^{\alpha + 3\beta +1}\big ) \\&\qquad \qquad \qquad \quad \times (\mathcal {K}+ N)(\mathcal {N}_{>N^{\alpha }/3}+1). \end{aligned}\end{aligned}$$

Here, an inequality of the form $\Sigma _{j\alpha } \le \mathcal {L}$ for a non-negative self-adjoint operator $\mathcal {L}$ refers to the statement that $\Sigma _{j\alpha }(\xi )\le \langle \xi ,\mathcal {L}\,\xi \rangle$, for all $\xi \in L^2_s(\Lambda ^N)$. In order to illustrate more explicitly how to bound the above terms, consider for example $\Sigma _{1a}$: Here, we bound

$$\begin{aligned} \begin{aligned} \Sigma _{1a}&\le \sum _{\begin{array}{c} r\in \Lambda ^*_+, p,q, s, t\in \Lambda ^*:\\ |p|,|q|,|s|,|t|\le N^\beta \\ |p+q-r|, |s+t-r|> N^\alpha /3 \end{array}} \!\!\! \Vert a_p a_q a_{s+t-r}\cdot \Vert \Vert a_{p+q-r}a_{s}a_{t}\cdot \Vert \\&\le \left( \sum _{\begin{array}{c} r\in \Lambda ^*_+, p,q, s, t\in \Lambda ^*:\\ |p|,|q|,|s|,|t|\le N^\beta \\ |p+q-r|, |s+t-r|> N^\alpha /3 \end{array}} \!\!\! \Big (\frac{|p|+1}{|s|+1}\Big )^2 \Vert a_p a_q a_{s+t-r}\cdot \Vert ^2 \right) ^{1/2}\\&\quad \times \left( \sum _{\begin{array}{c} r\in \Lambda ^*_+, p,q, s, t\in \Lambda ^*:\\ |p|,|q|,|s|,|t|\le N^\beta \\ |p+q-r|, |s+t-r|> N^\alpha /3 \end{array}} \!\!\! \Big (\frac{|s|+1}{|p|+1}\Big )^2\Vert a_{p+q-r}a_{s}a_{t}\cdot \Vert ^2\right) ^{1/2}\\&\le CN^{4\beta +1} (\mathcal {K}+ N) (\mathcal {N}_{>N^{\alpha }/3}+1). \end{aligned} \end{aligned}$$

The remaining contributions can be controlled in the same way, except the term $\Sigma _{3b}$: In this case, all momenta appearing in the creation operators are high, and in order to efficiently use the kinetic energy, we bound this term in a more involved way by

$$\begin{aligned} \Sigma _{3b}\le & {} \!\!\!\sum _{\begin{array}{c} r\in \Lambda ^*_+, p,q, s, t\in \Lambda ^*: p,q,s,t,\\ |q|> N^\beta , |s|, |t| \le N^\beta ,\\ |p|, |s+t-r|> N^\alpha /3 \end{array}} \!\!\! \frac{|s|+1}{|t|+1}\Vert (\mathcal {N}_s+1)^{\frac{1}{2}} a_p a_{s+t-r}\cdot \Vert \frac{|t|+1}{|s|+1}\Vert (\mathcal {N}_q+1)^{\frac{1}{2}} a_{p+q-r}a_{t}\cdot \Vert \\\le & {} \left( \!\!\!\sum _{\begin{array}{c} r\in \Lambda ^*_+, p,q, s, t\in \Lambda ^*: p,q,s,t,\\ |q|> N^\beta , |p+q-r|, |s|, |t| \le N^\beta ,\\ |p|, |s+t-r|> N^\alpha /3 \end{array}} \!\!\! \Big (\frac{|s|+1}{|t|+1}\Big )^2 \Big (\Vert \mathcal {N}_s^{\frac{1}{2}} a_p a_{s+t-r}\cdot \Vert ^2 +\Vert a_p a_{s+t-r}\cdot \Vert ^2\Big ) \right) ^{1/2}\\{} & {} \times \left( \!\!\!\sum _{\begin{array}{c} r\in \Lambda ^*_+, p,q, s, t\in \Lambda ^*: p,q,s,t,\\ |q|> N^\beta , |p+q-r|, |s|, |t| \le N^\beta ,\\ |p|, |s+t-r|> N^\alpha /3 \end{array}} \!\!\! \Big (\frac{|t|+1}{|s|+1}\Big )^2 \Big (\Vert \mathcal {N}_q^{\frac{1}{2}} a_{p+q-r}a_{t}\cdot \Vert ^2 +\Vert a_{p+q-r}a_{t}\cdot \Vert ^2 \Big ) \right) ^{1/2}\\{} & {} \quad +\left( \!\!\!\sum _{\begin{array}{c} r\in \Lambda ^*_+, p,q, s, t\in \Lambda ^*: p,q,s,t,\\ |q|, |p+q-r|> N^\beta , |s|, |t| \le N^\beta ,\\ |p|, |s+t-r|> N^\alpha /3 \end{array}} \!\!\! \Big (\frac{|s|+1}{|t|+1}\Big )^2 \Big (\Vert \mathcal {N}_s^{\frac{1}{2}} a_p a_{s+t-r}\cdot \Vert ^2 +\Vert a_p a_{s+t-r}\cdot \Vert ^2\Big ) \right) ^{1/2}\\{} & {} \quad \times \left( \!\!\!\sum _{\begin{array}{c} r\in \Lambda ^*_+, p,q, s, t\in \Lambda ^*: p,q,s,t,\\ |q|,|p+q-r|> N^\beta , |s|, |t| \le N^\beta ,\\ |p|, |s+t-r|> N^\alpha /3 \end{array}} \!\!\! \Big (\frac{|t|+1}{|s|+1}\Big )^2 \Big (\Vert \mathcal {N}_q^{\frac{1}{2}} a_{p+q-r}a_{t}\cdot \Vert ^2 +\Vert a_{p+q-r}a_{t}\cdot \Vert ^2 \Big ) \right) ^{1/2}\\{} & {} \le \big ( N^{\frac{3}{2}\alpha \!+\! \frac{5}{2}\beta \!+\!\frac{1}{2}} (\mathcal {N}_{>N^{\beta }}+N^{3\alpha })^{\frac{1}{2}} \! +\! N^{3\alpha +\beta } (\mathcal {N}_{>N^{\beta }}+N^{3\alpha })\big ) (\mathcal {K}+ \!N \!+\! N^{5\beta }) (\mathcal {N}_{>N^{\alpha }/3}\!+\!1), \end{aligned}$$

where we set $\mathcal {N}_s:=a_{s}^* a_{s}$.

Collecting the above estimates and multiplying by a factor $N^{2\kappa -2\alpha -2}$, we arrive at

$$\begin{aligned}\begin{aligned}&N^{2\kappa -2\alpha -2} \!\!\!\!\!\!\!\!\!\sum _{\begin{array}{c} r\in \Lambda ^*_+, p,q, s, t\in \Lambda ^*:\\ (p+q-r,r),(s+t-r,r)\in ( \text {P}_{\text {L}} ^2)^c,\\ (p,q),(s,t)\in \text {P}_{\text {L}} ^2 \end{array}} \!\!\! |\langle \xi , a_p^* a_q^* a_{s+t-r}^* a_{p+q-r}a_{s}a_{t}\xi \rangle |\\&\quad \le CN^{2\kappa -2\alpha -2} \langle \xi ,\big (N^{4\alpha } (\mathcal {N}_{>N^{\beta }}+N^{3\alpha }) + N^{\frac{5}{2}\alpha + \frac{3}{2}\beta +\frac{1}{2}} (\mathcal {N}_{>N^{\beta }}+N^{3\alpha })^{\frac{1}{2}} +N^{\frac{3}{2}\alpha +\frac{5}{2}\beta +1}\big )\\&\qquad \times (\mathcal {K}+ N + N^{5\beta }) (\mathcal {N}_{>N^{\alpha }/3}+1)\xi \rangle . \end{aligned} \\ \end{aligned}$$

$$\square $$

Before concluding Theorem 1, the last ingredient that we need is some mild a priori information on the energy of the ground state vector $\psi _N$, as remarked around Eq. (9).

### Lemma 5

Let $\psi _N$ denote the normalized ground state vector of $H_N$, defined in (1), and let $\beta \ge 0$. Then, $\psi _N$ satisfies the a priori bounds

$$\begin{aligned}\begin{aligned}&\langle \psi _N, \mathcal {N}_{> N^{\beta }}\psi _N\rangle \le C N^{1+\kappa -2\beta }, \\&N^{-1}\langle \psi _N, \mathcal {K}\mathcal {N}_{> N^{\beta }}\psi _N\rangle \le C N^{1+2\kappa -2\beta } + C N^{\frac{3}{2}\kappa +\frac{1}{2}\beta }, \\&N^{-2}\langle \psi _N, \mathcal {K}\mathcal {N}^2_{> N^{\beta }}\psi _N\rangle \le C N^{1+3\kappa -4\beta } + CN^{\beta +2\kappa } +CN^{\frac{5}{2}\kappa -\frac{3}{2}\beta }. \end{aligned}\end{aligned}$$

### Proof

The first bound is a direct consequence of (9) and the fact that $E_N \le CN^{1+\kappa }$. For the bound on $\mathcal {K}\mathcal {N}_{\ge N^{\beta }}$, we use a commutator argument as in [1, 6, 7]. We bound

$$\begin{aligned}\begin{aligned} N^{-1}\langle \psi _N, \mathcal {K}\mathcal {N}_{> N^{\beta }}\psi _N\rangle&\le N^{-1}\langle \psi _N, \mathcal {K}\psi _N\rangle ^{\frac{1}{2}}\langle \psi _N, \mathcal {N}_{> N^{\beta }} \mathcal {K}\mathcal {N}_{> N^{\beta }}\psi _N\rangle ^{\frac{1}{2}}\\&\le C N^{\frac{\kappa }{2}-\frac{1}{2}} \langle \psi _N, \mathcal {N}_{> N^{\beta }} \mathcal {K}\mathcal {N}_{> N^{\beta }}\psi _N\rangle ^{\frac{1}{2}} \end{aligned}\end{aligned}$$

and then

$$\begin{aligned}\begin{aligned}&\frac{1}{N}\langle \psi _N, \mathcal {N}_{> N^{\beta }}\mathcal {K}\mathcal {N}_{> N^{\beta }}\psi _N\rangle \\&\quad \le \frac{1}{N}\langle \psi _N, \mathcal {N}_{> N^{\beta }} H_N \mathcal {N}_{> N^{\beta }}\psi _N\rangle \\&\quad = \frac{E_N}{N} \langle \psi _N, \mathcal {N}^2_{> N^{\beta }}\psi _N\rangle + \frac{1}{N}\langle \psi _N, \mathcal {N}_{> N^{\beta }}\big [ H_N, \mathcal {N}_{> N^{\beta }}\big ]\psi _N\rangle \\&\quad \le CN^{\kappa -2\beta }\langle \psi _N, \mathcal {K}\mathcal {N}_{> N^{\beta }}\psi _N\rangle + \frac{1}{N}\langle \psi _N, \mathcal {N}_{> N^{\beta }}\big [ H_N, \mathcal {N}_{> N^{\beta }}\big ]\psi _N\rangle . \end{aligned}\end{aligned}$$

To estimate the commutator contribution on the r.h.s. in the previous equation, we write

$$\begin{aligned}H_N -\mathcal {K}= \frac{1}{2}\int _{\Lambda ^2}dxdy\, N^{2-2\kappa }V(N^{1-\kappa }(x-y))\check{a}_x^*\check{a}_y^*\check{a}_x\check{a}_y=: \mathcal {V}_N,\end{aligned}$$

where $\check{a}_x:=\sum _{p\in \Lambda ^*} e^{ipx}a_p$ denotes the usual operator valued distribution annihilating a particle at $x\in \Lambda$, and we note $[\mathcal {K}, \mathcal {N}_{> N^{\beta }}]=0$ as well as $[\mathcal {V}_N, \mathcal {N}_{> N^{\beta }}]=[\mathcal {N}_{\le N^{\beta }}, \mathcal {V}_N]$ with

$$\begin{aligned} \begin{aligned} \big [ \mathcal {N}_{\le N^{\beta }}, \mathcal {V}_N\big ] = \sum _{p\in \Lambda ^*: |p|\le N^{\beta }} \int _{\Lambda ^2}dxdy\, N^{2-2\kappa }V(N^{1-\kappa }(x-y))e^{ipx}\check{a}_p^*\check{a}_y^*\check{a}_x\check{a}_y + \text {h.c.}\ \end{aligned} \end{aligned}$$

Now, basic Cauchy–Schwarz estimates imply that

$$\begin{aligned}\begin{aligned}&N^{-1}\big |\big \langle \psi _N, \mathcal {N}_{> N^{\beta }}\big [ \mathcal {N}_{\le N^{\beta }}, \mathcal {V}_N\big ]\psi _N\big \rangle \big |\\&\quad \le CN^{-1}\sum _{p\in \Lambda ^*: |p|\le N^{\beta }} \int _{\Lambda ^2}dxdy\,N^{2-2\kappa }V(N^{1-\kappa }(x-y)) \Vert a_p \check{a}_y \,\mathcal {N}_{> N^{\beta }} \psi _N\Vert \Vert \check{a}_x\check{a}_y\psi _N\Vert \\&\qquad +CN^{-1}\sum _{p\in \Lambda ^*: |p|\le N^{\beta }} \int _{\Lambda ^2}dxdy\,N^{2-2\kappa }V(N^{1-\kappa }(x-y)) \Vert a_p \check{a}_y \psi _N\Vert \Vert \check{a}_x\check{a}_y \,\mathcal {N}_{> N^{\beta }}\psi _N\Vert \\&\quad \le C N^{\frac{\beta }{2}+\frac{\kappa }{2}-1}\big ( \Vert (\mathcal {K}+a^*_0a_0)^{\frac{1}{2}}\mathcal {N}_{> N^{\beta }}\psi _N \Vert \Vert \mathcal {V}_N^{1/2}\psi _N\Vert \\ {}&\qquad + \Vert (\mathcal {K}+a^*_0a_0)^{\frac{1}{2}}\psi _N \Vert \Vert \mathcal {V}_N^{1/2}\mathcal {N}_{> N^{\beta }}\psi _N\Vert \big ) \\&\quad \le C N^{\frac{\beta }{2}+ \kappa -\frac{1}{2}} \Vert H_N^{\frac{1}{2}}\mathcal {N}_{> N^{\beta }}\psi _N \Vert +C N^{\frac{\beta }{2}+ \kappa }\Vert \mathcal {N}_{> N^{\beta }}\psi _N\Vert . \end{aligned} \end{aligned}$$

Combining the previous estimates with $ab\le \frac{a^2}{2}+\frac{b^2}{2}$, we conclude

$$\begin{aligned}\begin{aligned}&\frac{1}{N}\langle \psi _N, \mathcal {N}_{> N^{\beta }} H_N \mathcal {N}_{> N^{\beta }}\psi _N\rangle \\&\quad \le C N^{\kappa -2\beta }\langle \psi _N, \mathcal {K}\mathcal {N}_{> N^{\beta }}\psi _N\rangle + C N^{\frac{\beta }{2}+ \kappa -\frac{1}{2}} \Vert H_N^{\frac{1}{2}}\mathcal {N}_{> N^{\beta }}\psi _N \Vert +C N^{\frac{\beta }{2}+ \kappa }\Vert \mathcal {N}_{> N^{\beta }}\psi _N\Vert \\&\quad \le C N^{\kappa -2\beta }\langle \psi _N, \mathcal {K}\mathcal {N}_{> N^{\beta }}\psi _N\rangle + CN^{\beta +2\kappa } + \frac{1}{2N}\langle \psi _N, \mathcal {N}_{> N^{\beta }} H_N \mathcal {N}_{> N^{\beta }}\psi _N\rangle \end{aligned}\end{aligned}$$

and therefore

$$\begin{aligned}\frac{1}{N}\langle \psi _N, \mathcal {N}_{> N^{\beta }} H_N \mathcal {N}_{> N^{\beta }}\psi _N\rangle \le C N^{\kappa -2\beta }\langle \psi _N, \mathcal {K}\mathcal {N}_{> N^{\beta }}\psi _N\rangle + CN^{\beta +2\kappa }. \end{aligned}$$

As a consequence, we obtain that

$$\begin{aligned}\begin{aligned}&N^{-1}\langle \psi _N, \mathcal {K}\mathcal {N}_{> N^{\beta }}\psi _N\rangle \le C N^{1+2\kappa -2\beta } + C N^{\frac{3}{2}\kappa +\frac{1}{2}\beta }, \\&N^{-2}\langle \psi _N, \mathcal {N}_{> N^{\beta }} \mathcal {K}\mathcal {N}_{> N^{\beta }}\psi _N\rangle \le C N^{1+3\kappa -4\beta } + CN^{\beta +2\kappa } +CN^{\frac{5}{2}\kappa -\frac{3}{2}\beta }. \end{aligned}\end{aligned}$$

$$\square $$

We are now ready to prove our main result.

### Proof of Theorem 1

Let $$ \psi _N$$ denote the normalized ground state vector of $$ H_N$$, given some parameter $$ \kappa \in [0,\frac{1}{20})$$. Let $$ \text {P}_{\text {L}} $$ be defined as in (3) and choose

$$\begin{aligned} \alpha := (1+\epsilon )\frac{41}{10}\kappa \end{aligned}$$

for some sufficiently small $\epsilon >0$; in particular $\alpha \in [0,1-\kappa ]$. Now, by (4), we have that

$$\begin{aligned}\begin{aligned} \langle \psi _N, H_N\psi _N\rangle&\ge \sum _{r\in \Lambda _+^*}|r|^2 \langle \psi _N, c^*_r c_r \psi _N\rangle - \langle \psi _N, R_N\psi _N\rangle \\&\quad +\frac{N^{\kappa }}{2N}\!\!\!\sum _{\begin{array}{c} p,q,r\in \Lambda ^*: \\ p,q, p+r, q-r \in \text {P}_{\text {L}} \end{array}}\!\!\!\!\! \langle \varphi _{p+r}\otimes \varphi _{q-r}, V_{\text {ren}}\varphi _p\otimes \varphi _q \rangle \langle \psi _N, a^*_{p+r}a^*_{q-r}a_pa_q\psi _N\rangle \end{aligned}\end{aligned}$$

and our goal is to estimate the terms on the right-hand side. We start with the kinetic energy term. Combining the bounds from Lemmas 2 and 5, we find that

$$\begin{aligned}\begin{aligned}&\sum _{r\in \Lambda _+^*}|r|^2 \langle \psi _N, c^*_r c_r \psi _N\rangle -4\pi ^2 \langle \psi _N, \mathcal {N}_+ \psi _N\rangle \\&\quad \ge - C N^{1+\kappa -2 \delta } - CN^{1+3\kappa +\frac{1}{2}\delta -\frac{7}{2}\alpha } - CN^{\frac{5}{2}\kappa +\frac{1}{2}\delta -\alpha } = o(N), \end{aligned}\end{aligned}$$

where we used (9), the choice $\frac{\kappa }{2}< \delta < \alpha$ and the identity $\mathcal {N}_{< N^{\delta }}- a^*_0a_0 = \mathcal {N}_+-\mathcal {N}_{\ge N^{\delta }}$.

Proceeding similarly for the remaining error terms, we obtain from Lemma 3 that

$$\begin{aligned}\begin{aligned}&\frac{N^{\kappa }}{2N}\!\!\!\sum _{\begin{array}{c} p,q,r\in \Lambda ^*: \\ p,q, p+r, q-r \in \text {P}_{\text {L}} \end{array}}\!\!\!\!\! \langle \varphi _{p+r}\otimes \varphi _{q-r}, V_{\text {ren}}\varphi _p\otimes \varphi _q \rangle \langle \psi _N, a^*_{p+r}a^*_{q-r}a_pa_q\psi _N\rangle \\&\quad \ge 4\pi \mathfrak {a}N^{1+\kappa } - C N^{1+2\kappa -2\alpha } - CN^{\kappa +3\alpha } =4\pi \mathfrak {a}N^{1+\kappa }+o(N) \end{aligned}\end{aligned}$$

and from Lemma 4, assuming $\beta = (1+\epsilon )\frac{5}{2}\kappa$ for sufficiently small $\epsilon >0$, that

$$\begin{aligned}\begin{aligned} |\langle \psi _N, R_N\psi _N\rangle |&\le C N^{1+5\kappa -2\beta } + C N^{\frac{1}{2}+\frac{9}{2}\kappa +\frac{5}{2}\alpha -2\beta } + C N^{1+\frac{9}{2}\kappa +\frac{1}{2}\beta -\frac{3}{2}\alpha }\\&\quad +C N^{\frac{1}{2}+4\kappa +\alpha +\frac{1}{2}\beta }+C N^{1+4\kappa +\frac{5}{2}\beta -\frac{5}{2}\alpha } \\&= o(N) + C N^{\frac{1}{2}+\frac{5}{2}\alpha -\frac{1}{2}\kappa +O(\epsilon ) } + C N^{\frac{1}{2}+\frac{21}{4}\kappa +\alpha +O(\epsilon ) } = o(N). \end{aligned}\end{aligned}$$

Combining this with the ground state energy upper bound $E_N\le 4\pi \mathfrak {a}N^{1+\kappa }+o(N)$, as pointed out in the second remark after Theorem 1, we get

$$\begin{aligned}\begin{aligned} 4\pi \mathfrak {a}N^{1+\kappa }+o(N)&\ge \langle \psi _N, H_N\psi _N\rangle \ge 4\pi \mathfrak {a}N^{1+\kappa } + 4\pi ^2 \langle \psi _N, \mathcal {N}_+\psi _N\rangle +o(N) \end{aligned}\end{aligned}$$

and thus conclude that

$$\begin{aligned}\lim _{N\rightarrow \infty }N^{-1} \langle \psi _N, \mathcal {N}_+\psi _N\rangle =\lim _{N\rightarrow \infty } \big (1- \langle \varphi _0, \gamma _N^{(1)}\varphi _0\rangle \big )=0. \end{aligned}$$

$$\square $$

## A Proof of the Bounds (7) and (8)

### Proof of the Bounds (7) and (8)

Throughout this appendix, we assume $p,q,s,t\in \Lambda ^*$ and we abbreviate $\langle \mathcal {L}\rangle _{pq, st}:=\langle \varphi _{p}\otimes \varphi _{q}, \mathcal {L}\,\varphi _{s}\otimes \varphi _{t} \rangle$ for every operator $\mathcal {L}$ on $L^2(\Lambda ^2)$. Let us begin with a few elementary observations: it is clear that the operator $N^{1-\kappa }V_N$ preserves the total momentum and that

$$\begin{aligned}\langle N^{1-\kappa }V_N \rangle _{pq,st}\!\le \! \delta _{p+q, s+t}\!\int _{\Lambda ^2} dx_1dx_2\, N^{3-3\kappa } V(N^{1-\kappa }(x_1-x_2))\!\le \! \delta _{p+q, s+t}\Vert V\Vert _1. \end{aligned}$$

Combining this with the fact that $-\Delta _{x_1}-\Delta _{x_2}+V_N$ and hence its pseudo-inverse

$$\begin{aligned} \mathcal {R}:=\Pi _{\text {H}} \big [ \Pi _{\text {H}} ( -\Delta _{x_1}-\Delta _{x_2}+V_N)\Pi _{\text {H}} \big ]^{-1} \Pi _{\text {H}} \end{aligned}$$

from $\Pi _{\text {H}} L^{2}(\Lambda ^2)$ to $\Pi _{\text {H}} L^{2}(\Lambda ^2)$ also preserve the total momentum, we get that

$$\begin{aligned}\begin{aligned} \langle V_{\text {ren}}\rangle _{pq,st}&\le C\delta _{pq,st}\sup _{p,q\in \Lambda ^*} \Big ( 1+N^{1-\kappa }\big \langle V_N^{1/2} \Pi _{\text {H}} V_N^{1/2} \mathcal {R}V_N^{1/2}\Pi _{\text {H}} V_N^{1/2} \big \rangle _{pq,pq} \\&\quad + N^{1-\kappa }\big \langle V_N^{1/2} \Pi _{\text {L}} V_N^{1/2} \mathcal {R}V_N^{1/2}\Pi _{\text {L}} V_N^{1/2} \big \rangle _{pq,pq}\Big ). \end{aligned} \end{aligned}$$

To control the right-hand side, we make use of the operator inequalities

$$\begin{aligned} 0\le \Pi _{\text {H}} V_N^{1/2} \mathcal {R}V_N^{1/2}\Pi _{\text {H}} \le 1 \;\text { and }\; \mathcal {R}\le N^{-2\alpha }\Pi _{\text {H}} \le N^{-2\alpha }. \end{aligned}$$

This implies on the one hand that

$$\begin{aligned} N^{1-\kappa }\big \langle V_N^{1/2} \Pi _{\text {H}} V_N^{1/2} \mathcal {R}V_N^{1/2}\Pi _{\text {H}} V_N^{1/2} \big \rangle _{pq,pq}\le \langle N^{1-\kappa }V_N \rangle _{pq,pq} = \Vert V\Vert _1 \end{aligned}$$

and on the other hand that

$$\begin{aligned} N^{1-\kappa }\big \langle V_N^{1/2} \Pi _{\text {L}} V_N^{1/2} \mathcal {R}V_N^{1/2}\Pi _{\text {L}} V_N^{1/2} \big \rangle _{pq,pq} \le N^{-2\alpha } \Vert V\Vert _1 \Vert \Pi _{\text {L}} V_N^{1/2}\varphi _p\otimes \varphi _q \Vert ^2_{\infty }.\end{aligned}$$

Looking at the Fourier expansion

$$\begin{aligned} \Pi _{\text {L}} V_N^{1/2}\varphi _p\otimes \varphi _q = \sum _{s\in \text {P}_{\text {L}} :p+q-s\in \text {P}_{\text {L}} } N^{2\kappa -2} \widehat{V^{1/2}}((s-p)/N^{1-\kappa })\varphi _{s}\otimes \varphi _{p+q-s}, \end{aligned}$$

the assumptions that $V\in L^1(\mathbb {R}^3)$ having compact support and that $\alpha \le 1-\kappa$ imply that $\Vert \Pi _{\text {L}} V_N^{1/2}\varphi _p\otimes \varphi _q \Vert _{\infty }\le CN^{\alpha }$ so that altogether $|\langle V_{\text {ren}}\rangle _{pq,st}|\le C \delta _{pq,st}$.

Next, let us switch to the second bound in (7). We first show that

13 $$\begin{aligned} |\langle V_{\text {ren}}\rangle _{00,00}-8\pi \mathfrak {a}|\le CN^{\alpha +\kappa -1}. \end{aligned}$$

Up to minor modifications, this bound follows as in [16, Appendix A], so let us focus on the key steps. Denote by *f* the zero energy scattering solution in $\mathbb {R}^3$ such that

$$\begin{aligned} (-2\Delta + V)f=0 \end{aligned}$$

with $\lim _{|x|\rightarrow \infty } f(x) = 1$. It is well-known (see e.g. [30, Appendix C]) that $0\le f\le 1$, that *f* is radial and that for $x\in \mathbb {R}^3$ outside the support of *V*, we have that $f(x) = 1-\mathfrak {a} /|x|.$ Moreover, a basic integration by parts shows that

$$\begin{aligned} 8\pi \mathfrak {a} = \int _{\mathbb {R}^3} dx\, V(x)f(x) = \int _{\Lambda }dx\, N^{3-3\kappa }(Vf)(N^{1-\kappa }x). \end{aligned}$$

Let us denote $w:= 1- f$ which is easily seen to satisfy the bounds

$$\begin{aligned} w(x) \le \frac{C}{|x|}, \hspace{0.5cm} |\widehat{w} (p)|\le \frac{C}{1+|p|^2} \end{aligned}$$

for some constant $C>0$ (e.g. based on the identity $w = (-2\Delta )^{-1} Vf$). Moreover, pick a smooth bump function $\chi \in C^{\infty }_c( B_{1/2}(0))$ such that $\chi (x) =1$ if $|x|\le \frac{1}{4}$ and define

$$\begin{aligned}(x_1,x_2) \mapsto \phi _N(x_1-x_2):= \chi (x_1-x_2)w(N^{1-\kappa }(x_1-x_2)) \in L^2(\Lambda ^2).\end{aligned}$$

By slight abuse of notation, we identify $\phi _N$ with the associated multiplication operator in $L^2(\Lambda ^2)$. As explained in [16], we then have the identity

$$\begin{aligned} \mathcal {R}V_N \varphi _0\otimes \varphi _0 = \phi _N \varphi _0\otimes \varphi _0 + \mathcal {R}\zeta _N \varphi _0\otimes \varphi _0 - (1-\mathcal {R}V_N)\Pi _{\text {L}} \phi _N\varphi _0\otimes \varphi _0, \end{aligned}$$

where $$ \zeta _N(x_1-x_2):=N^{\kappa -1}\zeta (x_1-x_2)$$ for

$$\begin{aligned}x\mapsto \zeta (x):=2\mathfrak {a}\frac{ (\Delta \chi )(x)}{|x|} -4\mathfrak {a}\frac{ (\nabla \chi )(x)\cdot (x)}{|x|^3}\in C^{\infty }_{0}\big (B_{1/2}(0)\cap \overline{B}^c_{1/4}(0)\big ). \end{aligned}$$

Using that $8\pi \mathfrak {a} = N^{1-\kappa }\langle V_N, (1-\phi _N)\rangle _{00,00}$, this yields

$$\begin{aligned} \langle V_{\text {ren}}\rangle _{00,00}= 8\pi \mathfrak {a} + N^{1-\kappa }\langle \mathcal {R}V_N, \zeta _N\rangle _{00,00} + N^{1-\kappa }\langle V_N, (1-\mathcal {R}V_N)\Pi _{\text {L}} \phi _N\rangle _{00,00}. \end{aligned}$$

Now observe that for $|p|>N^{\alpha }$, we have that

14 $$\begin{aligned} \begin{aligned} \langle \mathcal {R}V_N\rangle _{-pp,00}&= \frac{-\langle V_N\mathcal {R}V_N\rangle _{-pp,00}}{2|p|^{2}} +\frac{\langle V_N-\Pi _{\text {L}} (1-V_N\mathcal {R}) V_N\rangle _{-pp,00}}{2|p|^{2}}\\&= \frac{\langle V_N(1-\mathcal {R}V_N)\rangle _{-pp,00}}{2|p|^{2}}\end{aligned}\end{aligned}$$

and otherwise $\langle \mathcal {R}V_N\rangle _{-pp,00}=0$ (by definition of $\mathcal {R}$) s.t. $N^{1-\kappa }\langle \mathcal {R}V_N, \zeta _N\rangle _{00,00}\le C N^{\kappa -1}$. Similarly, $|\widehat{\phi }_N(p)|\le CN^{\kappa -1}(1+|p|^2)^{-1}$ and Cauchy-Schwarz imply that

$$\begin{aligned}\begin{aligned}&| N^{1-\kappa }\langle V_N, (1-\mathcal {R}V_N)\Pi _{\text {L}} \phi _N\rangle _{00,00} |\\&\quad \le C \Vert V\Vert _1\big ( \Vert \Pi _{\text {L}} \phi _N\Vert _\infty +N^{-\alpha }\Vert \Pi _{\text {L}} V_N^{1/2}\Pi _{\text {L}} \phi _N\Vert _\infty \big )\le C N^{\alpha +\kappa -1}. \end{aligned} \end{aligned}$$

Combining the previous estimates yields (13). In fact, using $N^{1-\kappa }\langle V_N(1-\phi _N)\rangle _{p+r\, q-r,pq} = \widehat{Vf}(r/N^{1-\kappa })$, we can also compute $\langle V_{\text {ren}}\rangle _{00,00}$ to higher precision and obtain that

$$\begin{aligned} \begin{aligned} \langle V_{\text {ren}}\rangle _{00,00}&= 8\pi \mathfrak {a} +\frac{N^{\kappa -1}}{2}\sum _{\begin{array}{c} 0\ne s\in \text {P}_{\text {L}} \end{array}} \frac{(8\pi \mathfrak {a})^2}{|s|^2} +O(N^{\kappa -1}) +O(N^{2\kappa +2\alpha -2}). \end{aligned} \end{aligned}$$

We omit the details as the second term is irrelevant for our range of $\kappa$, it only becomes relevant if one wants to consider the Lee–Huang–Yang order.

To get (7), we combine (13) with two further steps. On the one hand, we have that

15 $$\begin{aligned} |\langle V_{\text {ren}}\rangle _{pq,st}- \langle V_{\text {ren}}\rangle _{(p+q)0,(s+t)0}|\le CN^{\kappa -1} \big ( |p|+ |q|+|s|+|t|\big ) \end{aligned}$$

whenever $$ p+q = s+t $$. This bound follows very similarly as the first bound in (7): Since $$ V_N$$ is a multiplication operator, (15) clearly holds if we replace $$ V_{\text {ren}}$$ by $$ N^{1-\kappa }V_N$$. Hence, it is enough to prove (15) for $$ V_{\text {ren}}$$ replaced by $$ N^{1-\kappa }V_N \mathcal {R}V_N $$. In this case, we write

$$\begin{aligned}\begin{aligned}&N^{1-\kappa }\langle V_N \mathcal {R}V_N\rangle _{pq,st}- N^{1-\kappa }\langle V_N \mathcal {R}V_N\rangle _{(p+q)0,(s+t)0}\\&\quad = N^{1-\kappa } \langle \varphi _p\otimes \varphi _q, V_N \mathcal {R}V_N ( \varphi _{s}\otimes \varphi _{t} - \varphi _{s+t}\otimes \varphi _{0} ) \rangle \\&\qquad + N^{1-\kappa }\langle ( \varphi _{p}\otimes \varphi _{q} - \varphi _{p+q}\otimes \varphi _{0} ), V_N \mathcal {R}V_N \varphi _{s+t}\otimes \varphi _{0} \rangle . \end{aligned}\end{aligned}$$

Now, given any pair $k,l \in \Lambda ^*$, a direct computation shows that

$$\begin{aligned}\begin{aligned} N^{1-\kappa } \Vert V_N^{1/2}( \varphi _{k}\otimes \varphi _{l} - \varphi _{k+l}\otimes \varphi _{0} ) \Vert ^2&= 2 \widehat{V}(0) - 2 \widehat{V}(l/N^{1-\kappa })\le \frac{ C |l|^2 }{N^{2-2\kappa } }. \end{aligned} \end{aligned}$$

Note that the last step follows from a second-order Taylor expansion and the fact that $(\nabla _p \widehat{V}(./N^{1-\kappa } )(0) = N^{2-2\kappa } \int _{\mathbb {R}^3} dx\, (-ix) V(x) =0$, *V* being radial. Similarly, we get

$$\begin{aligned}\begin{aligned}&N^{-2\alpha } \Vert \Pi _{\text {L}} V_N^{1/2}( \varphi _{k}\otimes \varphi _{l} - \varphi _{k+l}\otimes \varphi _{0} ) \Vert ^2_{\infty } \\&\quad = N^{-2\alpha } \Big \Vert \sum _{\begin{array}{c} s\in \text {P}_{\text {L}} :\\ k+l-s\in \text {P}_{\text {L}} \end{array}} \!\!\!\!\!\!N^{2\kappa -2}\big ( \widehat{V^{1/2}}((s-k)/N^{1-\kappa }) - \widehat{V^{1/2}}((s-k-l)/N^{1-\kappa })\big ) \varphi _{s}\otimes \varphi _{k+l-s}\Big \Vert _\infty ^2\\&\quad \le C|l|^2/N^{2-2\kappa }. \end{aligned}\end{aligned}$$

Proceeding now as in the proof of the first bound in (7), we obtain (15).

Combining (15) with (13), the second bound in (7) thus follows if we prove that

16 $$\begin{aligned} |\langle V_N\mathcal {R}V_N \rangle _{p0,p0}- \langle V_N\mathcal {R}V_N \rangle _{00,00}|\le CN^{2\kappa -\alpha -2} |p|^2 \end{aligned}$$

for every $p\in \Lambda ^*_+$. This can be proved similarly as detailed in [16, Appendix A]: Define

$$\begin{aligned} -\Delta ^{(p)}:= (-i\nabla _{x_1}+p)^2 -\Delta _{x_2}, \hspace{0.5cm} \mathcal {R}^{(p)}:= \Pi _{\text {H}} ^{+} \big [\Pi _{\text {H}} ^{+} (-\Delta ^{(p)}+V_N)\Pi _{\text {H}} ^{+}\big ]^{-1}\Pi _{\text {H}} ^{+}, \end{aligned}$$

where the orthogonal projection $\Pi _{\text {H}} ^{+}$ maps onto

$$\begin{aligned} \overline{\text {span}\{ \varphi _k\otimes \varphi _l: (k,l)\in ( \text {P}_{\text {L}} ^2)^c \text { and }l\ne 0 \} }.\end{aligned}$$

Notice that this ensures $\langle \xi , -\Delta ^{(p)}\xi \rangle \ge 4\pi ^2$ for every $\xi \in \Pi _{\text {H}} ^{+}L^2(\Lambda ^2)$, by construction of the projection $\Pi _{\text {H}} ^{+}$. In particular, $\mathcal {R}^{(p)}$ is well-defined. Now, based on the observation

$$\begin{aligned}\langle V_N\mathcal {R}V_N \rangle _{p0,p0} = \langle V_N e^{-ipx_1}\mathcal {R}e^{ipx_1} V_N \rangle _{00,00} \end{aligned}$$

and the fact that $-\Delta ^{(p)} = e^{-ipx_1}(-\Delta _{x_1}-\Delta _{x_2})e^{ipx_1}$ for $p\in \Lambda ^*$, it follows that

$$\begin{aligned}\begin{aligned}&\langle V_N e^{-ipx_1}\mathcal {R}e^{ipx_1} V_N\rangle _{00,00}-\langle V_N\mathcal {R}^{(p)}V_N\rangle _{00,00} \\&\quad = -\langle V_N \mathcal {R}^{(p)}e^{-ipx_1}\Pi _{\text {L}} (1-V_N\mathcal {R})V_N\rangle _{00,p0} +\langle V_N (1-\mathcal {R}^{(p)}V_N)\Pi _{\text {L}} ^{+} e^{-ipx_1}\mathcal {R}V_N\rangle _{00,p0}. \end{aligned}\end{aligned}$$

Here, we set $\Pi _{\text {L}} ^{+}:= 1- \Pi _{\text {H}} ^{+}$. Since $V_N \mathcal {R}^{(p)}$ preserves the total momentum and projects onto a subset of $( \text {P}_{\text {L}} ^2)^c$, we have that

$$\begin{aligned}\begin{aligned}&\langle V_N \mathcal {R}^{(p)}e^{-ipx_1}\Pi _{\text {L}} (1-V_N\mathcal {R})V_N\rangle _{00,p0} \\&\quad = \sum _{q\in \text {P}_{\text {L}} ^c\cap \Lambda ^*_+; \,s,t\in \text {P}_{\text {L}} } \langle V_N \mathcal {R}^{(p)}\rangle _{00,-qq} \langle \varphi _{-q}\otimes \varphi _q, \varphi _{s-p}\otimes \varphi _t\rangle \langle (1-V_N\mathcal {R})V_N\rangle _{st,p0}=0. \end{aligned}\end{aligned}$$

On the other hand, using that $\mathcal {R}= \Pi _{\text {H}} \mathcal {R}$ so that

$$\begin{aligned}\begin{aligned} \langle \mathcal {R}V_N\rangle _{(p-q)q, p0}&= \frac{\langle V_N- V_N\mathcal {R}V_N\rangle _{(p-q)q, p0}}{|p-q|^2 + |q|^2}-\frac{\langle \Pi _{\text {L}} (1-V_N\mathcal {R})V_N\rangle _{(p-q)q, p0}}{|p-q|^2 + |q|^2} \\&= \frac{\langle V_N- V_N\mathcal {R}V_N\rangle _{(p-q)q, p0}}{|p-q|^2 + |q|^2} \end{aligned} \end{aligned}$$

if $(p-q,q)\in ( \text {P}_{\text {L}} ^2)^c$ and $\langle \mathcal {R}V_N\rangle _{(p-q)q, p0} =0$ otherwise, we obtain that

$$\begin{aligned}\begin{aligned}&\big \Vert \Pi _{\text {L}} ^{+} e^{-ipx_1} \mathcal {R}V_N e^{ipx_1}\big \Vert _{\infty } \le \sum _{s\in \text {P}_{\text {L}} } | \langle \mathcal {R}V_N\rangle _{(p-s)s, p0}|\le CN^{\alpha +\kappa -1},\\&\big \Vert \Pi _{\text {L}} ^{+} V_N^{1/2}\Pi _{\text {L}} ^{+} e^{-ipx_1} \mathcal {R}V_N e^{ipx_1}\big \Vert _{\infty } \le \sum _{s,t\in \text {P}_{\text {L}} } \frac{C}{N^{3-3\kappa }(1+|t|^2)} \le CN^{2\alpha +\kappa -1}. \end{aligned}\end{aligned}$$

Hence, arguing similarly as in the previous steps, we find that

$$\begin{aligned}\begin{aligned}&|\langle V_N (1-\mathcal {R}^{(p)}V_N)\Pi _{\text {L}} ^{+} e^{-ipx_1}\mathcal {R}V_N\rangle _{00,p0}|\\ {}&\quad \le \frac{CN^{\kappa }}{N}\big ( \big \Vert \Pi _{\text {L}} ^{+} e^{-ipx_1} \mathcal {R}V_N e^{ipx_1}\big \Vert _{\infty }+N^{-\alpha }\big \Vert \Pi _{\text {L}} ^{+} V_N^{1/2} \Pi _{\text {L}} ^{+} e^{-ipx_1} \mathcal {R}V_N e^{ipx_1}\big \Vert _{\infty } \big )\\ {}&\quad \le C N^{\alpha +2\kappa -2}. \end{aligned}\end{aligned}$$

Notice here that we used additionally the operator inequalities $-\Delta ^{(p)} \ge N^{2\alpha }\Pi _{\text {H}} ^+$ and, as a consequence, $\mathcal {R}^{(p)} \le N^{-2\alpha }\Pi _{\text {H}} ^+\le N^{-2\alpha }$ in the image

$$\begin{aligned} \Pi _{\text {H}} ^+ \big ({\textbf {1}}_{\text {P} = 0}\,L^2(\Lambda ^2)\big ) =\Pi _{\text {H}} ^{+}\, \overline{\text {span}\{ \varphi _s\otimes \varphi _{-s}:s\in \Lambda ^*\}} = \overline{\text {span}\{ \varphi _s\otimes \varphi _{-s}:|s| > N^{\alpha }\}} \end{aligned}$$

of the space of zero total momentum $\text {P}:=-i\nabla _{x_1}-i\nabla _{x_2}$ under $\Pi _{\text {H}} ^+$, and that both

$$\begin{aligned} V_N^{1/2}\Pi _{\text {L}} ^{+}V_N^{1/2} \Pi _{\text {L}} ^{+} e^{-ipx_1}\mathcal {R}V_N e^{ipx_1}\in L^2(\Lambda ^2)\;\; \text { and } \;\;V_N^{1/2}\Pi _{\text {L}} ^{+}V_N^{1/2}\in L^2(\Lambda ^2) \end{aligned}$$

have zero total momentum.

Collecting the previous bounds, proving (16) reduces to proving that

$$\begin{aligned}|\langle V_N\mathcal {R}^{(p)} V_N \rangle _{00,00}- \langle V_N\mathcal {R}V_N \rangle _{00,00}|\le CN^{2\kappa -\alpha -2} |p|^2.\end{aligned}$$

To show this, we use that

$$\begin{aligned} | \langle \mathcal {R}^{(sp)}V_N \rangle _{-qq,00}| = \frac{|\langle V_N-V_N\mathcal {R}^{(sp)}V_N\rangle _{-qq,00}|}{|sp-q|^2+|q|^2}\le \frac{CN^{\kappa -1}}{|sp-q|^2+|q|^2} \end{aligned}$$

for all $s\in [0,1]$ and $|q|>N^{\alpha }$ (otherwise $\langle \mathcal {R}^{(sp)}V_N \rangle _{-qq,00}=0$). Together with

$$\begin{aligned} \langle V_N\mathcal {R}^{(p)} V_N \rangle _{00,00} = \langle V_N\mathcal {R}^{(-p)} V_N \rangle _{00,00} \end{aligned}$$

and a second-order Taylor expansion, we find that

$$\begin{aligned}|\langle V_N\mathcal {R}^{(p)} V_N \rangle _{00,00}- \langle V_N\mathcal {R}V_N \rangle _{00,00}|\le CN^{2\kappa -2} |p|^2\int _{|q|> N^{\alpha }}dq\, |q|^{-4}\le N^{2\kappa -\alpha -2} |p|^2, \end{aligned}$$

which implies (16) and thus (7).

Finally, Eq. (8) is a direct consequence of the identity $(-\Delta _{x_1}-\Delta _{x_2}) \eta = \Pi _{\text {H}} V_{\text {ren}}\Pi _{\text {L}}$ and the bound (7) implying that $( |p|^2+|q|^2) | \langle \eta \rangle _{pq,st}| \le C \delta _{pq,st} {\textbf {1}}_{( \text {P}_{\text {L}} ^2)^c}((p,q)){\textbf {1}}_{ \text {P}_{\text {L}} ^2}((s,t))$. $\square$

## Footnotes

1 It is worth to point out that the arguments of [28], which build on energy bounds from [31, 32], can in fact be used to prove BEC in the parameter range $\kappa \in [0,\frac{1}{10})$; see also [30, Chapter 7].

2 To be more precise, the localization procedure of [21] replaces the standard Laplacian in the periodic setting by a more involved localized kinetic energy operator, see [21, Eq. (2.7)] For a recent overview that focuses on the key steps of the energy bounds in the simpler translation invariant setting, see [22].

## References

[1] Adhikari, A., Brennecke, C., Schlein, B.: Bose–Einstein condensation beyond the Gross–Pitaevskii regime. Ann. Henri Poincaré **22**, 1163–1233 (2021) 33786012 10.1007/s00023-020-01004-1 PMC7956940

[2] Basti, G., Cenatiempo, S., Olgiati, A., Pasqualetti, G., Schlein, B.: A second order upper bound for the ground state energy of a hard-sphere gas in the Gross–Pitaevskii regime. Commun. Math. Phys. **399**, 1–55 (2023)

[3] Basti, G., Cenatiempo, S., Schlein, B.: A new second-order upper bound for the ground state energy of dilute Bose gases. Forum Math. Sigma **9**, e74. 10.1017/fms.2021.66 (2021)

[4] Benedikter, N., de Oliveira, G., Schlein, B.: Quantitative derivation of the Gross–Pitaevskii equation. Commun. Pure Appl. Math. **64**(8), 1399–1482 (2014)

[5] Boccato, C., Brennecke, C., Cenatiempo, S., Schlein, B.: Complete Bose–Einstein condensation in the Gross–Pitaevskii regime. Commun. Math. Phys. **359**(3), 975–1026 (2018)

[6] Boccato, C., Brennecke, C., Cenatiempo, S., Schlein, B.: The excitation spectrum of Bose gases interacting through singular potentials. J. Eur. Math. Soc. **22**(7), 2331 (2020)

[7] Boccato, C., Brennecke, C., Cenatiempo, S., Schlein, B.: Bogoliubov theory in the Gross–Pitaevskii limit. Acta Math. **222**(2), 219–335 (2019)

[8] Boccato, C., Brennecke, C., Cenatiempo, S., Schlein, B.: Optimal rate for Bose–Einstein condensation in the Gross–Pitaevskii regime. Commun. Math. Phys. **376**, 1311–1395 (2020)

[9] Boccato, C., Seiringer, R.: The Bose gas in a box with Neumann boundary conditions. Ann. Henri Poincaré **24**(5), 1505–1560 (2023)

[10] Bogoliubov, N.N.: On the theory of superfluidity. Izv. Akad. Nauk. USSR **11**, 77 (1947). (Engl. Transl. J. Phys. (USSR) **11**, 23 (1947))

[11] Brennecke, C., Caporaletti, M., Schlein, B.: Excitation spectrum for Bose Gases beyond the Gross–Pitaevskii regime. Rev. Math. Phys. **34**(09), 2250027 (2022)

[12] Brennecke, C., Schlein, B.: Gross–Pitaevskii dynamics for Bose–Einstein condensates. Anal. PDE **12**(6), 1513–1596 (2019)

[13] Brennecke, C., Schlein, B., Schraven, S.: Bose–Einstein condensation with optimal rate for trapped Bosons in the Gross–Pitaevskii regime. Math. Phys. Anal. Geom. **25**, 12 (2022)

[14] Brennecke, C., Schlein, B., Schraven, S.: Bogoliubov theory for trapped Bosons in the Gross–Pitaevskii regime. Ann. Henri Poincaré **23**, 1583–1658 (2022)

[15] Brietzke, B., Fournais, S., Solovej, J.P.: A simple 2nd order lower bound to the energy of dilute Bose Gases. Commun. Math. Phys. **376**, 323–351 (2020)

[16] Brooks, M.: Diagonalizing Bose Gases in the Gross–Pitaevskii regime and beyond. Preprint: arXiv:2310.11347 10.1007/s00220-024-05199-w PMC11655615 39711825

[17] Caraci, C., Cenatiempo, S., Schlein, B.: Bose–Einstein condensation for two dimensional Bosons in the Gross–Pitaevskii regime. J. Stat. Phys. (2021). 10.1007/s10955-021-02766-6 10.1007/s10955-021-02766-6 PMC8550145 34720183

[18] Caraci, C., Cenatiempo, S., Schlein, B.: The excitation spectrum of two-dimensional Bose Gases in the Gross–Pitaevskii regime. Ann Henri Poincaré **24**, 2877 (2023) 37424535 10.1007/s00023-023-01278-1 PMC10328896

[19] Caraci, C., Olgiati, A., Saint Aubin, D., Schlein, B.: Third order corrections to the ground state energy of a Bose gas in the Gross–Pitaevskii regime. Preprint: arXiv:2311.07433 10.1007/s00220-025-05320-7 PMC12134057 40474986

[20] Dyson, F.J.: Ground-state energy of a hard-sphere gas. Phys. Rev. **106**, 20–26 (1957)

[21] Fournais, S.: Length scales for BEC in the dilute Bose gas. In: Partial Differential Equations, Spectral Theory, and Mathematical Physics. The Ari Laptev Anniversary Volume. EMS Series of Congress Reports, vol. 18, pp. 115-133 (2021). 10.4171/ECR/18-1/7

[22] Fournais, S., Girardot, T., Junge, L., Morin, L., Olivieri, M.: Lower bounds on the energy of the Bose gas. Reviews in Mathematical Physics. 10.1142/S0129055X23600048 10.1007/s00220-023-04907-2 PMC11231024 38983883

[23] Fournais, S., Solovej, J.P.: The energy of dilute Bose gases. Ann. Math. **192**(3), 893–976 (2020)

[24] Fournais, S., Solovej, J.P.: The energy of dilute Bose gases II: the general case. Invent. Math. **232**, 863–994 (2023)

[25] Haberberger, F., Hainzl, C., Nam, P.T., Seiringer, R., Triay, A.: The free energy of dilute Bose gases at low temperatures. Preprint: arXiv:2304.02405

[26] Hainzl, C.: Another proof of BEC in the GP-limit. J. Math. Phys. **62**, 051901 (2021)

[27] Hainzl, C., Schlein, B., Triay, A.: Bogoliubov theory in the Gross–Pitaevskii limit: a simplified approach. Forum Math., Sigma **10**, e90. 10.1017/fms.2022.78 (2022)

[28] Lieb, E.H., Seiringer, R.: Proof of Bose–Einstein condensation for dilute trapped gases. Phys. Rev. Lett. **88**, 170409 (2002) 12005741 10.1103/PhysRevLett.88.170409

[29] Lieb, E.H., Seiringer, R.: Derivation of the Gross–Pitaevskii equation for rotating Bose gases. Commun. Math. Phys. **264**(2), 505–537 (2006)

[30] Lieb, E.H., Seiringer, R., Solovej, J.P., Yngvason, J.: The Mathematics of the Bose Gas and its Condensation. Oberwolfach Seminars, vol. 34. Birkhäuser Verlag, Basel (2005)

[31] Lieb, E.H., Seiringer, R., Yngvason, J.: Bosons in a trap: a rigorous derivation of the Gross–Pitaevskii energy functional. Phys. Rev. A **61**, 043602 (2000)

[32] Lieb, E.H., Yngvason, J.: Ground state energy of the low density Bose gas. Phys. Rev. Lett. **80**, 2504–2507 (1998)

[33] Nam, P.T., Napiórkowski, M., Ricaud, J., Triay, A.: Optimal rate of condensation for trapped bosons in the Gross–Pitaevskii regime. Anal. PDE **15**(6), 1585–1616 (2022)

[34] Nam, P.T., Ricaud, J., Triay, A.: The condensation of a trapped dilute Bose gas with three-body interactions. Probab. Math. Phys. **4**, 91–149 (2023)

[35] Nam, P.T., Rougerie, N., Seiringer, R.: Ground states of large bosonic systems: the Gross–Pitaevskii limit revisited. Anal. PDE **9**(2), 459–485 (2016)

[36] Nam, P.T., Triay, A.: Bogoliubov excitation spectrum of trapped Bose gases in the Gross–Pitaevskii regime. J. Math. Pures Appl. **176**, 18–101 (2023)

[37] Yau, H.-T., Yin, J.: The second order upper bound for the ground state energy of a Bose gas. J. Stat. Phys. **136**(3), 453–503 (2009)

